# Functional SARS-CoV-2-specific T cells of donor origin in allogeneic stem cell transplant recipients of a T-cell-replete infusion: A prospective observational study

**DOI:** 10.3389/fimmu.2023.1114131

**Published:** 2023-03-03

**Authors:** Corinna La Rosa, Flavia Chiuppesi, Yoonsuh Park, Qiao Zhou, Dongyun Yang, Ketevan Gendzekhadze, Minh Ly, Jing Li, Teodora Kaltcheva, Sandra Ortega Francisco, Miguel-Angel Gutierrez, Haris Ali, Salman Otoukesh, Idoroenyi Amanam, Amandeep Salhotra, Vinod A. Pullarkat, Ibrahim Aldoss, Michael Rosenzweig, Ahmed M. Aribi, Anthony S. Stein, Guido Marcucci, Sanjeet Singh Dadwal, Ryotaro Nakamura, Stephen J. Forman, Monzr M. Al Malki, Don J. Diamond

**Affiliations:** ^1^ Department of Hematology and Hematopoietic Cell Transplantation, City of Hope, Duarte, CA, United States; ^2^ Histocompatibility Laboratory, Department of Hematology and Hematopoietic Cell Transplantation, City of Hope, Duarte, CA, United States; ^3^ Department of Infectious Disease, City of Hope, Duarte, CA, United States

**Keywords:** SARS-CoV-2 T cells, hematopoietic cell transplant, memory phenotype, CD137^+^ T cells, IFN-γ response

## Abstract

In the current post-pandemic era, recipients of an allogeneic hematopoietic stem cell transplant (HCT) deserve special attention. In these vulnerable patients, vaccine effectiveness is reduced by post-transplant immune-suppressive therapy; consequently, severe acute respiratory syndrome coronavirus 2 (SARS-CoV-2) disease (COVID-19) is often associated with elevated morbidity and mortality. Characterizing SARS-CoV-2 adaptive immunity transfer from immune donors to HCT recipients in the context of immunosuppression will help identify optimal timing and vaccination strategies that can provide adequate protection to HCT recipients against infection with evolving SARS-CoV-2 variants. We performed a prospective observational study (NCT04666025 at ClinicalTrials.gov) to longitudinally monitor the transfer of SARS-CoV-2-specific antiviral immunity from HCT donors, who were either vaccinated or had a history of COVID-19, to their recipients *via* T-cell replete graft. Levels, function, and quality of SARS-CoV-2-specific immune responses were longitudinally analyzed up to 6 months post-HCT in 14 matched unrelated donor/recipients and four haploidentical donor/recipient pairs. A markedly skewed donor-derived SARS-CoV-2 CD4 T-cell response was measurable in 15 (83%) recipients. It showed a polarized Th1 functional profile, with the prevalence of central memory phenotype subsets. SARS-CoV-2-specific IFN-γ was detectable throughout the observation period, including early post-transplant (day +30). Functionally experienced SARS-CoV-2 Th1-type T cells promptly expanded in two recipients at the time of post-HCT vaccination and in two others who were infected and survived post-transplant COVID-19 infection. Our data suggest that donor-derived SARS-CoV-2 T-cell responses are functional in immunosuppressed recipients and may play a critical role in post-HCT vaccine response and protection from the fatal disease.

**Clinical trial registration:**

clinicaltrials.gov, identifier NCT04666025.

## Introduction

Intense immunosuppression exacerbates life-threatening sequelae and adverse outcomes of severe acute respiratory syndrome coronavirus 2 (SARS-CoV-2) disease (COVID-19) in patients receiving allogeneic hematopoietic cell transplantation (HCT), a curative therapy for many hematological diseases (1–3). In addition, patients with hematological malignancy have varying degrees of immune dysfunction pre- and post-HCT, and those who develop COVID-19 often have poor survival outcomes, including and exceeding a high mortality rate (4–6). Vaccination substantially decreases morbidity and mortality in HCT recipients at high risk for a variety of infections post-HCT (7). However, despite the success of FDA- and EMA-approved vaccination programs in healthy adult and pediatric populations, the efficacy of COVID-19 licensed vaccines is limited in these immunocompromised patients (8–12) and can be further reduced by frequently evolving SARS-CoV-2 variants (13). Nonetheless, delaying immunizations against SARS-CoV-2 until complete immune reconstitution is achieved can markedly increase the risk of COVID-19 complications in these vulnerable patients (14). Consequently, clinical studies to determine strategies for increasing early vaccine response and to identify alternative therapeutic platforms or optimal timing for post-HCT vaccinations are of high priority (15).

It has been shown by us and others that donor B- and T-cell immunity from pathogen exposure or vaccination pre-graft collection can be transferred to the recipient, expanding during immune reconstitution and improving vaccination responses and/or controlling post-HCT infections (16, 17). In recipients with a successful HCT outcome, circulating T cells arise from donor CD34^+^ cells during the first year post-HCT and can react to antigens exposed to the donor through natural infection or vaccination before transplantation (18, 19). Therefore, donor pathogen exposure or vaccination pre-graft collection can be beneficial to the recipient when the mounting cellular and humoral response augments immune reconstitution and controls post-HCT natural infection or increase vaccination responses (20). Though B-cell functional reconstitution and adaptive humoral immune recovery after HCT is delayed (21), the critical role that T-cell responses may play in mitigating SARS-CoV-2 infection, including modulating disease severity (22) has been reported in HCT patients (23, 24). During the current era of COVID-19 vaccination, there is a timely opportunity to assess the role of SARS-CoV-2-specific antiviral cellular immunity. Characterizing SARS-CoV-2 adaptive immunity transfer from immune donors to HCT recipients (16) in the context of immunosuppressive therapy by analyzing its levels, functionality, and quality during immune reconstitution can provide important insight to improve prophylaxis and treatment management (25).

In this framework, we designed a prospective observational study with the aim of longitudinally monitoring SARS-CoV-2-specific antiviral immunity transferred from HCT donors, who were either vaccinated or had a history of COVID-19, to their recipients *via* T-cell-replete graft infusion. The immune analysis panel included multiparameter flow-cytometry T-cell functional assays measuring 4-1BB (CD137) activation marker combined with a memory phenotype (26, 27) and enzyme-linked immunosorbent spot (ELISPOT) for IFN-γ detection (28). SARS-CoV-2-specific neutralization assays based on lentiviral pseudovirus and qualitative in-house developed ELISA were implemented to assess humoral immunity (28). Here, we report the outcome of the humoral and cellular immune monitoring analysis in HCT donor/recipient pairs, primarily focusing on the critical engraftment period through 6 months post-HCT.

## Materials and methods

### Study outline and oversight

This is an investigator-initiated, prospective, observational study of SARS-CoV-2 donor-recipient immunity transfer (NCT04666025 at ClinicalTrials.gov). The trial was approved on June 2020 by the City of Hope (COH) and the National Marrow Donor Program (NMDP) institutional review boards (IRBs) and was undertaken in accordance with Good Clinical Practice guidelines and the Declaration of Helsinki. NMDP and COH IRB-approved protocol permitted the participant’s enrollment, biospecimen collection, and immunological analyses. The 10/10 matched (with permissive HLA-DPB1 locus mismatch) unrelated donors (MUD), haploidentical (haplo) donors, and their HCT recipient (R) pairs gave written consent to participate in this study. MUDs from the US, Mexico, Europe, and Israel were enrolled through NMDP (haplo donors) at COH. Eligible HCT donors had to be either exposed to SARS-CoV-2 or fully vaccinated with a licensed COVID-19 vaccine before graft transfer. A total of 89 donors consented, and blood was drawn at least 30 days (d) before granulocyte colony-stimulating factor (GCSF) for peripheral blood CD34^+^ cell apheresis collection. All recipients consented and enrolled at COH, where they received a planned T-cell-replete allogeneic HCT. Levels, function, and quality of SARS-CoV-2-specific immunity were assessed pre-graft in the donors (single blood draw up to 30 days before GCSF administration for stem cell mobilization) and were longitudinally monitored in the recipient blood specimens collected at six time points (+d30, +d60, +d90, +d120, +d150, +d180) post-HCT. As per the study protocol design, HCT R specimens were not collected before the transplant. As per FDA and the guidelines for “COVID-19 Management in Hematopoietic Cell Transplantation and Cellular Therapy Recipients” (29), inpatients and outpatients at HCT R were routinely screened and monitored by PCR for SARS-CoV-2. However, SARS-CoV-2 routine serology testing was not required, as it is not recommended in patients with hematologic malignancy (29). Specimens from a cohort of healthy COH employees who did not have a COVID-19 infection history (*N* = 16) and were enrolled in the COH IRB 20720 observational study of SARS-CoV-2 adaptive immunity after COVID-19 licensed vaccine were evaluated as a retrospective reference cohort for the current study. Blood specimens from healthy COH employees were collected before the BNT162b2 mRNA COVID-19 vaccination and at 60, 90, and 180 days afterward.

### Blood specimens for immune monitoring

Peripheral blood mononuclear cells (PBMC) and serum from HCT donors and recipients were separated from blood following standard protocols and stored in liquid nitrogen (30). Thawed specimens were used to perform immune monitoring and characterization of adaptive SARS-CoV-2 immune responses. MUD specimens, obtained through NMDP were shipped overnight at room temperature to COH, for processing and storage. PBMC was not derived from MUD specimens if received >24 hours after collection. MUD019, MUD033, MUD036, MUD040, MUD041, MUD046, MUD050, and MUD053 blood specimens, drawn outside the US were received >24 h after collection; therefore, only serum was obtained from these donors. Due to poor cell viability, day 90 PBMC for R036, day 60 PBMC for R054, day 180 PBMC for R065, day 90 PBMC and days 150 and 180 PBMC and serum (terminally ill, in hospice care patient lost to follow-up) for R078, and days 30, 60, 120, and 150 PBMC for R088 were not available. Moreover, the first study blood draw (day 30 post-HCT) was not obtained for R036 and R040, who had a late enrollment due to health issues, and for R019, a single vial of PBMC was derived at all time points.

### HLA typing and chimerism

Both tests were done as a part of pre- and post-HCT routine clinical evaluation. Donor and recipient HLA typing was performed using Next-Generation Sequencing kits (Scisco Genetics, Seattle, Washington, United States). Measurements of donor chimerism were performed as per COH standard of care, following current guidelines on approximately +d30, +d100, and +d180 post-HCT, using quantitative PCR (GenDx, Utrecht, The Netherlands) from peripheral blood, bone marrow, or CD3 subset. CD3 was enriched using magnetic beads (Stemcell Technologies, Vancouver, British Columbia, Canada) prior to DNA isolation (31).

### Immune monitoring panel

SARS-CoV-2 spike (S)- and nucleocapsid (N)-specific cellular and humoral responses were longitudinally monitored in available blood specimens at multiple time points (+d30, +d60, +d90, +d120, +d150, +d180) during the first 6 months of post-HCT immune reconstitution.

#### Cellular immune assays

ELISPOT was performed by stimulating cryopreserved PBMC with S and N peptide pools (15 mers, 11 aa overlap, >70% purity, GenScript, Piscataway, New Jersey, United States) and using fluorospot plates coated with IFN-γ capture antibody (Mabtech, Cincinnati, Ohio, United States) (28). SARS-CoV-2-specific T cells were longitudinally monitored by measuring concentrations of CD3^+^CD4^+^ and CD3^+^CD8^+^ T cells expressing the 4-1BB (CD137) activation marker following 24 h stimulation with either S-15mer megapool (15 mers, 10 aa overlap, >70% purity megapool peptide library synthesized and lyophilized as previously reported (32) was kindly provided by A. Grifoni and A. Sette, La Jolla Institute for Immunology) (27) or N peptide pools (GenScript,Piscataway, New Jersey, United States), as previously detailed (28). PBMC for each time point were labeled and analyzed by fluorescence-activated cytometry (Gallios™, Beckman Coulter with Kaluza analysis software, Brea, CA, USA) (26). The lower limit of detection for CD137^+^ T cells was 0.02% or 0.1 cells/μl. When either SARS-CoV-2-specific CD3^+^CD8^+^CD137^+^ T-cell or CD3^+^CD4^+^CD137^+^ T-cell populations were ≥0.2%, further analysis for CD28 and CD45RA memory membrane markers was feasible (30). CD45RA^+^CD28^+^ cells were classified as naïve, CD45RA^−^CD28^+^ cells were classified as central memory (TCM), and CD28^−^ cells were classified as effector T cells. Within the effector T-cell group, two subpopulations were identified: CD45RA^−^CD28^−^ cells (TEM) and CD45RA^+^CD28^−^ effector “revertant” T cells, re-expressing the RA isoform of the CD45 surface marker (TEMRA) (26, 33). Further details regarding CD137 analysis, gating strategy, and memory phenotype data are provided in the Supplementary material (**Supplementary Table 1S**; **Supplementary Figure 1S**).

#### Humoral immune assays

SARS-CoV-2-specific IgG antibodies were measured in serum specimens by an in-house-developed ELISA (28). Our assay identifies SARS-CoV-2 antibodies specific for Spike (S) S1+S2 ectodomain and the S receptor-binding domain (RBD) that interacts with angiotensin-converting enzyme 2 (ACE2) on the surface of ACE2-positive cells and the N protein that is one of the first B-cell targets during the initial phase of the SARS-CoV-2 infection. Neutralizing antibodies were quantified as previously described (28) using SARS-CoV-2 pseudovirus based on Wuhan-Hu-1 S sequence with D614G modification.

### Statistical analysis

Descriptive statistics were used to analyze donor and patient characteristics. GraphPad Prism 9.4.1 was used to compare groups with the Mann–Whitney test, to calculate Spearman’s correlation coefficients and their *p*-values, and to derive correlation matrices. All tests were two-sided. The Loess scatter plots were made using the ggplot2 package in R (https://cran.r-project.org/web/packages/ggplot2/index.html).

## Results

### Characteristics of the study population

From September 2020 through October 2021, 14 MUD/R and four haplo D/R eligible pairs were enrolled in this observational study, as per the COH IRB 20153 protocol (NCT04666025). Details of demographic, clinical characteristics, pre-HCT treatment, COVID-19 licensed vaccinations, and COVID-19 history of the study D/R pairs are shown in **Table 1**. Ten HCT donors received the BNT162b2 mRNA COVID-19 vaccine (77%), two the mRNA-1273 COVID-19 vaccine (15%), and one the ChAdOx1-S/nCoV-19 (recombinant) vaccine (8%). Among HCT recipients, nine received the BNT162b2 mRNA COVID-19 vaccine (82%), one the mRNA-1273 COVID-19 vaccine (9%), and one the JNJ-78436735 COVID-19 vaccine (9%). Nine (50%) study recipients (R033, R036, R040, R041, R049, R054, R078, R086, R087) received a licensed COVID-19 vaccine within 4 months pre-HCT. All enrolled recipients engrafted early post-HCT, approximately by +d30 with full donor chimerism (>95%) (31). Two haplo recipients developed active COVID-19 infection, R086 on +d123 and R88 on +d82 post-HCT, respectively; both cleared the infection after 35 days. Two doses of the BNT162b2 mRNA COVID-19 vaccine were administered post-HCT to R029 (on +d112 and +d139) and R046 (on +d147 and +d169). MUD recipients R036 and R049 expired before the study ended, on +d136 and +d83 post-HCT, respectively. No MUD, haplo donor, or R discontinued the study for personal reasons or refused to donate blood during the multiple, per protocol, planned blood draws.

**Table 1:** Characteristics of the study HCT donor/recipient pairs.

	Total (*N* = 18)	
Recipient age at HCT (years)		
Median age (IQR; years)	59 (41–63)	
Female donor to male recipient		
Yes	4 (22)	
No	14 (78)	
Primary diagnosis at HCT		
Acute lymphoblastic leukemia	1 (5.5)	
Acute myeloid leukemia	11 (61)	
Multiple myeloma	1 (5.5)	
Myelodysplastic syndrome	2 (11)	
Myeloproliferative neoplasm	3 (17)	
Karnofsky performance score (at conditioning for HCT)		
80	5 (28)	
90	9 (50)	
100	4 (22)	
Conditioning regimen		
Reduced intensity	12 (67)	
Myeloablative	6 (33)	
Donor: COVID-19 vaccination status/COVID-19 history		
Vaccinated/no COVID-19 history	13 (72)
Not vaccinated/COVID-19 history	5 (28)
Recipient: COVID-19 vaccination status COVID-19 history		
Not vaccinated/no COVID-19 history	5 (28)
Not vaccinated/pre-HCT COVID-19 history	1 (5.5)
Not vaccinated/post-HCT COVID-19 history	1 (5.5)
Vaccinated pre-HCT/no COVID-19 history	7 (39)
Vaccinated pre- and post-HCT/no COVID-19 history	1 (5.5)
Vaccinated pre-HCT/post-HCT COVID-19 history	1 (5.5)
Vaccinated post-HCT/no COVID-19 history	2 (11)

HCT, hematopoietic stem cell transplant; IQR, interquartile range. Values are numbers of patients (percentages) unless otherwise indicated.

### SARS-CoV-2-specific and functional CD137^+^ T cells

Donor-derived S- and N-specific CD3^+^CD4^+^ and CD3^+^CD8^+^ T cells expressing CD137 marker of T-cell functional activation (34, 35) were measurable early post-HCT and expanded during immune reconstitution in 15 recipients (*N* = 18; 83%). They included 11 MUD recipients and four haplo recipients, respectively. As for the three MUD recipients in whom SARS-CoV-2-specific CD137^+^ T cells remained below or at the assay detection limit (0.1 T cells/µl: R036, R040, R049), potent suppression of T-cell proliferation and function resulted from treatment of acute graft-versus-host disease (aGVHD). In particular, two of these patients received high steroid doses (>1 mg/kg of prednisone equivalent) in combination with tocilizumab or ruxolitinib, and one received itacitinib (36). In contrast, the use of post-transplant cyclophosphamide (PTCy) (37) in the four haplo recipients did not seem to deplete or impair SARS-CoV-2-specific T cells.

**Figure 1:**
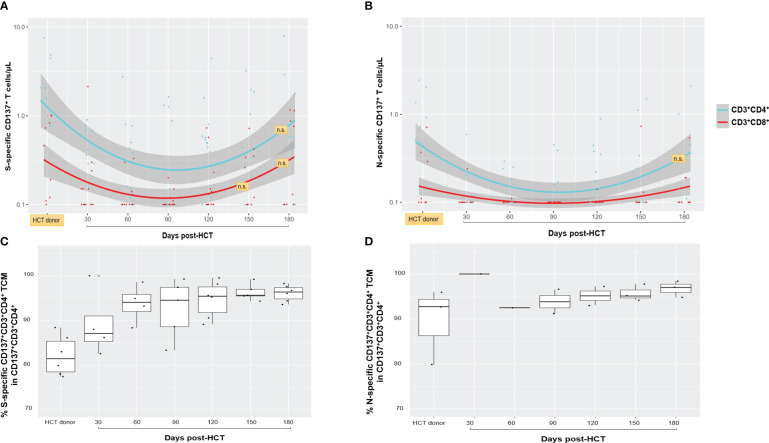
Spike (S)- and nucleocapsid (N)-specific and functional T-cell responses. **(A, B)** The frequency of S- and N-specific CD137^+^CD3^+^CD4^+^ (light blue lines and dots) and CD137^+^CD3^+^CD8^+^ (red lines and dots) are shown for nine HCT donors (for six MUD, no PBMC were derived as described in Material and methods; single blood collection at least 30 days before granulocyte colony-stimulating factor for peripheral blood CD34^+^ cell apheresis collection) and for 15 recipients who had detectable response above the assay detection limit (>0.1 T cells/µl), at the post-HCT time points indicated on the *x*-axis. The band shown in gray was computed using the Loess scatterplot smoother, providing the marginal geometric mean concentrations (95% confidence) through time for each T-cell subset. Individual measurement trajectories are shown for available PBMC specimens from each participant. T-cell concentrations after post-HCT COVID-19 vaccination or post-HCT COVID-19 diagnosis were not included. Differences between HCT donor and recipient T-cell levels were significant (*p* < 0.05, calculated by Mann–Whitney *U* test) unless otherwise indicated (n.s., not significant; orange boxes). N-specific CD137^+^CD3^+^CD8^+^ T-cell levels remained minimal or close to the assay detection limit in both HCT donors and recipients, and statistical comparison was not feasible. **(C, D)** Box plots showing percentages of S- **(C)** and N-specific **(D)** CD137^+^CD4^+^ TCM cells in the HCT donor and in the recipients at the post-HCT time points indicated on the *x*-axis. The box spans the interquartile range, the central bar shows the median, and the whiskers extend to 1.5 times the interquartile range. HCT, hematopoietic stem cell transplant; S, spike; N, nucleocapsid; TCM, central memory T cells.

As shown in **Figure 1** (upper Loess plots), S- (left plot) and N-specific T cells in the 15 responder recipients were characterized by a marked predominance of the CD4^+^ T-cell subset (38, 39). Only a minority of HCT donors had a history of COVID-19 infection (*N* = 5; 28%); therefore, the total frequency of N-specific CD137^+^ T cells was lower than S-specific CD137^+^ T cells. The N protein is one of the major immune targets during the early stage of SARS-CoV-2 infection, and it is generally recognized by individuals exposed to the virus (40). Nonetheless, the longitudinal immune profiles followed a consistently similar pattern for both S- and N-specific CD4^+^ and CD8 functionally activated T-cell subsets. The significantly lower concentrations of S- and N-specific CD4^+^ and CD8^+^ T cells observed post-HCT in the recipients compared to donors (*p* = 0.0002 by +d90 post-HCT for S-specific CD4^+^ T cells, calculated by Mann–Whitney *U* test) gradually increased during immune reconstitution. By +d150–180, the difference was no longer significant (*p* ≥ 0.05), and donors’ and recipients’ SARS-CoV-2-specific CD137^+^ T-cell levels were equal. Time points after post-HCT COVID-19 vaccination (R029 and R046) or COVID-19 diagnosis (R086 and R088) impacting T-cell frequency were not included in **Figure 1**. Nonetheless, the complete longitudinal profile for these patients is detailed in **Supplementary Figure 2S**.

In R086 and R088 patients who developed and survived COVID-19 infection post-HCT, both N- and S-specific T-cell responses were measurable, as shown in **Supplementary Figure 1S** upper plots. Elevated levels of S-specific CD4^+^ T cells were observed in R029 (>10 CD4^+^ T cells/µl max response) and R046 (~3 CD4^+^ T cells/µl max response) immediately following post-HCT BNT162b2 mRNA COVID-19 vaccination (**Supplementary Figure 2S**, lower plots). In the case of R029 (**Supplementary Figure 2S**, lower left plot), the magnitude of SARS-CoV-2-specific CD137^+^ T cells was 10 times higher than in their HCT donor on +d120 post-HCT, after administration of the COVID-19 vaccine first dose.

The memory phenotype profiles of S- and N-specific CD137^+^CD3^+^CD4^+^ T cells in HCT donors and recipients (**Figure 1**, bottom plots) were largely composed of experienced central memory T-cell subsets (CD45RA^−^CD28^+^, TCM) (26, 33, 41). In the recipients, they remained unchanged during the 6-month post-HCT observation period. Levels of SARS-CoV-2-specific CD137^+^CD3^+^CD8^+^ T cells ≥0.2% were infrequent; hence, a comparative analysis for memory membrane markers between HCT donors and recipients was not feasible (26).

### S- and N-specific IFN-γ production

**Figure 2:**
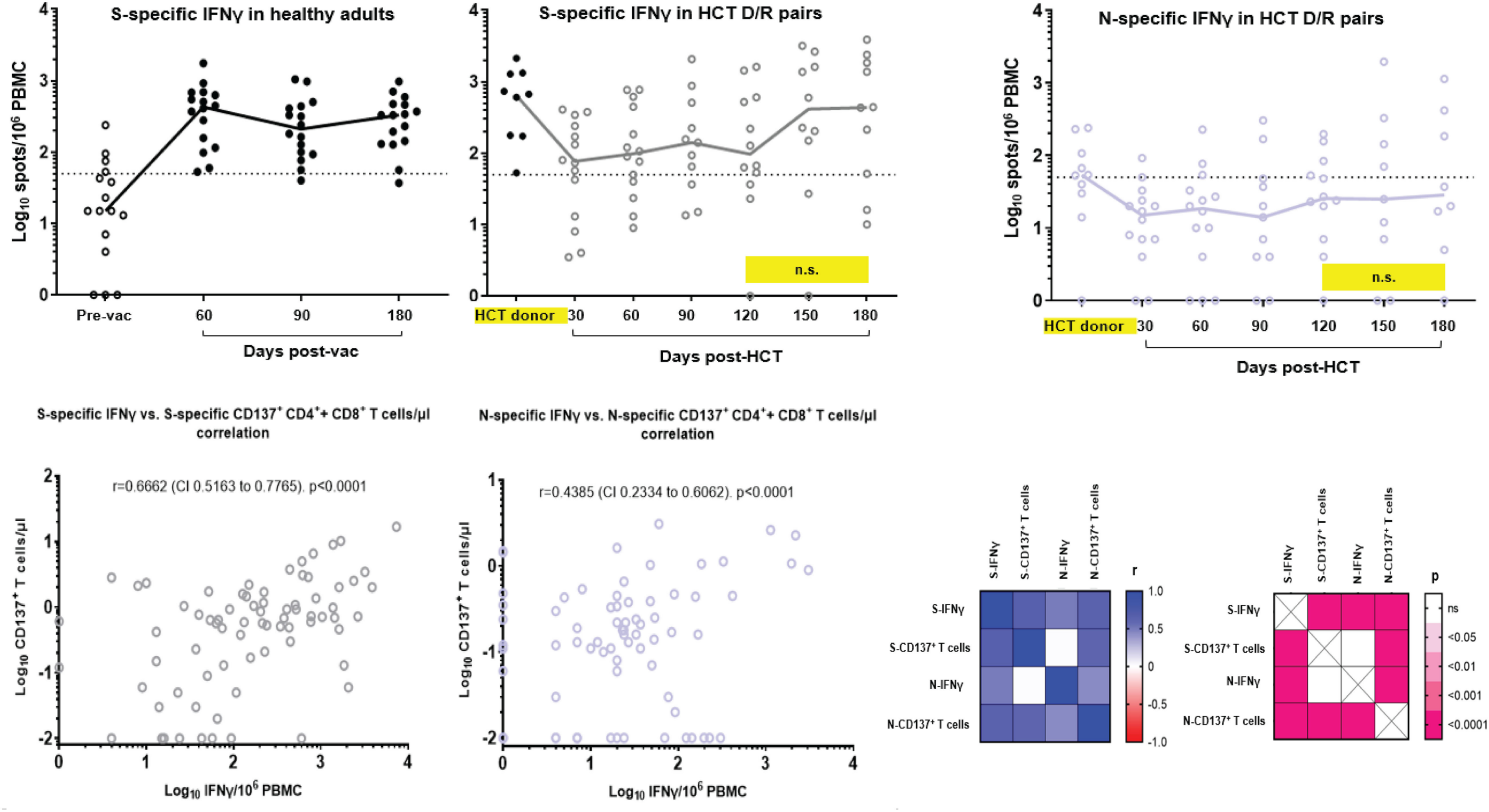
SARS-CoV-2-specific IFN-γ production and Th1 response correlative analyses. Upper panel, S-specific IFN-γ were measured by ELISPOT and expressed as spots/10^6^ PBMC in: *upper left plot*, a reference cohort of healthy adults (COH IRB 20720 observational study of SARS-CoV-2 adaptive immunity after COVID-19 licensed vaccines) before (empty dots) and after BNT162b2 mRNA COVID-19 vaccine, at the days indicated on the *x*-axis (filled black dots); *middle left plot*: HCT donor (filled black dots, single blood draw as specified in **Figure 1** legend) and recipient (grey empty dots) available PBMC specimens at the days post-HCT indicated on the *x*-axis. N-specific IFN-γ was measured by ELISPOT and expressed as spots/10^6^ PBMC in available PBMC from HCT donor and recipient pairs (purple empty dots, *upper right plot*). Black lines indicate median values; individual measurement trajectories are shown for each participant. In the upper middle and right plots, differences between HCT donor and recipient T-cell levels were significant (*p* < 0.05, calculated by Mann–Whitney *U* test) unless otherwise indicated (n.s., not significant; yellow boxes). The dashed lines represent the arbitrary threshold for positive response (50 spots/10^6^ PBMC). The total number of recipients analyzed for SARS-CoV-2-specific IFN-γ is N=14. R019 was not tested since the only PBMC vial obtained at each time point for this patient was used for the CD137 and memory phenotype assays (**Figure 1**). Bottom panel, Spearman’s correlation analysis between S-specific IFN-γ versus CD137^+^ T-cell (CD137^+^CD3^+^CD4^+^ + CD137^+^CD3^+^CD8^+^) levels (bottom left plot) and N-specific IFN-γ versus CD137^+^ T-cell (CD137^+^CD3^+^CD4^+^ + CD137^+^CD3^+^CD8^+^) levels (middle bottom plot). Spearman correlation coefficients and p values were calculated and plotted as a matrix (bottom right matrices). All available data obtained at all time points were used for the correlation analyses. Pre-vac, prevaccination; post-vac, post-vaccination; *r*, correlation coefficient; *CI*, confidence interval.

As mobilization of a polarized Th1 response with IFN-γ production is associated with protection from severe COVID-19 (42), we next longitudinally analyzed levels of SARS-CoV-2-specific IFN-γ by ELISPOT in PBMC of the study D/R pairs and in the reference cohort of healthy adult volunteers. In **Figure 2** (upper panel), the left plot reports S-specific IFN-γ production over time (with a follow-up of 6 months post-vaccination) in healthy adult volunteers who received a two-dose (each, 3 weeks apart) course of the BNT162b2 mRNA COVID-19 vaccine. The black dots in **Figure 2** (middle upper plot) show that levels of S-specific IFN-γ in PBMC from enrolled HCT donors were comparable to those observed in healthy volunteers post-vaccination. In the same plot, the grey dots show the longitudinal pattern of S-specific IFN-γ in the study recipients, post-HCT. The IFN-γ production profile from the recipients is characterized by a significant drop in the early post-HCT time points compared to the HCT donors (*p* = 0.0015 at +d30 post-HCT, calculated by the Mann–Whitney *U* test). A subsequent increase at later time points of post-HCT immune reconstitution results in S-specific IFN-γ levels becoming similar to those observed in the HCT donors. It is important to note that levels of IFN-γ in the study recipients who received a transplant from a donor who was either vaccinated or exposed to COVID-19 were significantly higher at all post-HCT time points (*p* = 0.0370 at +d30 post-HCT) than IFN-γ levels measured in healthy individuals before BNT162b2 mRNA COVID-19 vaccination. **Figure 2** (right upper plot) reports N-specific IFN-γ levels in the HCT D/R pairs, which show a trend analogous to S-specific IFN-γ.

Correlative analyses of S-specific IFN-γ versus CD137^+^ T-cell levels (**Figure 2** lower left plot) and N-specific IFN-γ versus CD137^+^ T-cell levels (**Figure 2** lower middle plot) revealed a highly significant association between measurements (*p* < 0.0001 for both S- and N-specific responses). The lower right panel of **Figure 2** details Spearman’s correlation coefficients and *p*-values calculated and plotted as matrices. The strong correlation of outcomes suggests that functionally active SARS-CoV-2-specific CD137^+^ T cells consistently displayed an IFN-γ Th1 response.

### SARS-CoV-2-specific humoral responses

**Figure 3:**
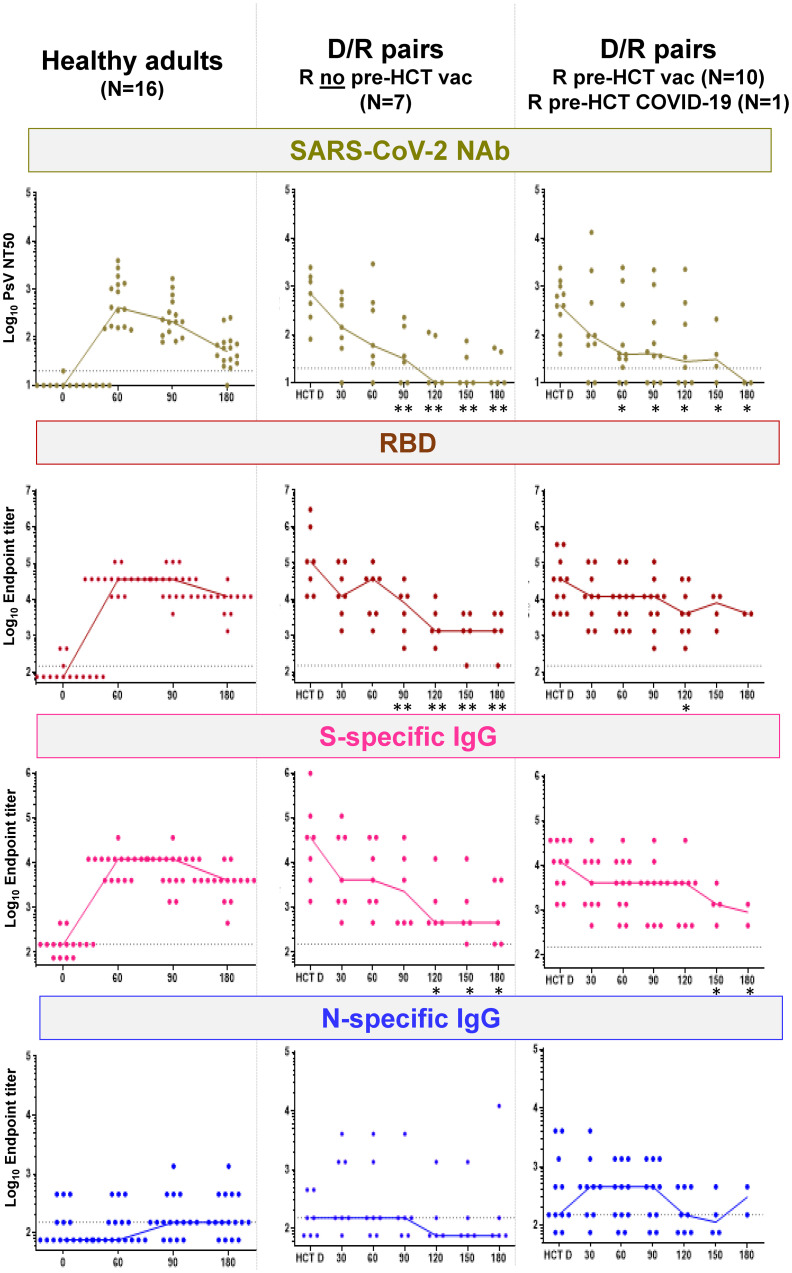
SARS-CoV-2-specific humoral responses. Left plots, SARS-CoV-2 NAb, RBD, and S- and N-specific IgG (as specified in the top plot banners) in healthy adults before BNT162b2 mRNA COVID-19 vaccine (reported as 0, in the *x*-axis), and afterwards at the time points shown in the *x*-axis (days 60, 90, and 180). Middle and right plots, SARS-CoV-2 NAb, RBD-, and S- and N-specific IgG (as specified in the top plot banners) for D/R pairs in whom R did not receive a licensed COVID-19 vaccine before HCT (*N* = 7; middle plots) and D/R pairs in whom R received a licensed COVID-19 vaccine before HCT (*N* = 10) or had a history of COVID-19 infection before HCT (*N* = 1; right plots). In the *x*-axis, HCT D indicates the donor response in serum obtained at least 30 days before granulocyte colony-stimulating factor for peripheral blood CD34^+^ cell apheresis collection; time points 30, 60, 90, 120, and 180 indicate the days post-HCT in which recipient serum specimens were analyzed. Star symbols on the post-HCT day indicate that differences in humoral responses (NAb, RBD, and S- and N-specific IgG) between HCT donors and recipients were significant (^*^ *p* < 0.05 and ^**^ *p* < 0.01, calculated by Mann–Whitney *U* test). SARS-CoV-2-neutralizing antibodies (NAb) are expressed on the log_10_ *y*-axis as serum dilution that neutralized 50% of the SARS-CoV-2 pseudovirus (PsV NT50). RBD and S- and N-specific IgG were measured using indirect ELISA and are expressed on the log_10_ *y*-axis as endpoint titers. Responses at time points after post-HCT COVID-19 vaccination, COVID-19 diagnosis, and administration of COVID-19 monoclonal antibody products were not included in the plots. Specifically, for R who did not receive a licensed COVID-19 vaccine before HCT (middle plots), the following are the sera tested at each time point: day 30, *N* = 7; day 60, *N* = 7; day 90, *N* = 6; day 120, *N* = 5; day 150, *N* = 5; and day 180, *N* = 5. For R who received a licensed COVID-19 vaccine before HCT or had a history of COVID-19 infection before HCT (right plots), the following are the sera tested at each time point: day 30, *N* = 9; day 60, *N* = 11; day 90, *N* = 10; day 120, *N* = 8; day 150, *N* = 4; and day 180, *N* = 2.

**Figure 3** summarizes SARS-CoV-2-specific humoral responses (lines indicate median values and dots individual measurements), including neutralization antibody (NAb) titers and IgG against the receptor-binding domain (RBD), S and N, in the reference cohort of healthy volunteers (left plots) and in the study D/R pairs (middle and right plots). The outcome of the humoral response for study recipients who did not receive a COVID-19 licensed vaccine pre-HCT and did not have a history of COVID-19 (*N* = 7) is shown in the middle plots of **Figure 3**. In the patients who were vaccinated before HCT with COVID-19 licensed vaccines (*N* = 10) and in the ones who had a history of pre-HCT COVID-19 infection, adaptive humoral immunity was of both donor and recipient origin. Hence, these patients are collectively shown in the right plots of **Figure 3**. Responses at time points after post-HCT COVID-19 vaccination, COVID-19 diagnosis, and administration of COVID-19 monoclonal antibody products are not included in **Figure 3** (D/R plots). All HCT donors had elevated NAb, RBD, and S-specific IgG, which were comparable to those measured in healthy adults after BNT162b2 mRNA COVID-19 vaccination. Levels of N-specific IgG were significantly higher in donors with a history of COVID-19 (*N* = 5; *p* = 0.0085 calculated by Mann–Whitney *U* test) compared to COVID-19-vaccinated donors (*N* = 13; **Supplementary Figure 3S**).

The post-HCT SARS-CoV-2-specific adaptive humoral response was clearly measurable in all recipients. In particular, there was no difference (*p* ≥ 0.05 by Mann–Whitney *U* test) in NAb, RBD, and S- and N-specific IgG titers between +d30 post-HCT and HCT donors. However, titers slowly declined through the end of the study in the recipients, persisting longer in those recipients who had been vaccinated with licensed COVID-19 vaccines or exposed to COVID-19 before HCT (**Figure 3**, right plots) compared to those who were not (**Figure 3**, middle plots). In particular, NAb responses became minimal around 3 months post-HCT, and N responses were detectable in a few recipients only. In contrast, RBD and S-specific IgG responses remained detectable until the end of the study.

In contrast, elevated NAb (**Supplementary Figure 1S**) was measured in recipients after both diagnoses of post-HCT COVID-19 infection (R086 and R088) and post-HCT BNT162b2 mRNA COVID-19 vaccination (R029 and R046). In particular, the R086 haplo recipient, who developed early post-HCT COVID-19 infection and who had been vaccinated before transplant (-d168 and -d174), showed remarkably high and sustained levels of NAb. In the two recipients who received a licensed COVID-19 vaccine post-HCT, both NAb and SARS-CoV-2-specific T-cell immunity greatly increased after the first dose.

## Discussion

We longitudinally characterized the transfer of SARS-CoV-2-specific immunity from COVID-19-vaccinated or COVID-19 previously infected MUD and haplo donors to T-cell-replete HCT recipients, who all achieved complete donor chimerism (31) around d+30 post-HCT. In the study cohort, we described the levels, quality, and functionality of donor-derived SARS-CoV-2-specific cellular and humoral responses. In recipients who developed and survived COVID-19 infection or were vaccinated post-HCT, both immunologic branches of donor-derived SARS-CoV-2 adaptive immunity were measurable and substantially increased in COVID-19-vaccinated patients. In the other study recipients who were neither infected nor vaccinated post-HCT, donor-derived SARS-CoV-2-specific, functional, and experienced memory T cells steadily expanded during the post-HCT immune reconstitution and eventually reached HCT donor levels. In contrast, SARS-CoV-2 adaptive humoral immunity kept decreasing during the 6-month post-HCT observation period, though in recipients who were vaccinated before HCT, modest antibody titers persisted.

To our knowledge, this is the first reported evidence of the possible protective role of donor-derived SARS-CoV-2 T cells in recipients who survived post-HCT COVID-19. This study outcome confirms that donor-derived SARS-CoV-2 T-cell-mediated responses can be preserved in immunosuppressed patients during immune-specific reconstitution and can increase COVID-19 vaccination effectiveness (12, 16, 24, 43), even in the presence of ongoing immunosuppressive treatment (5, 44).

Routine measurements of donor chimerism showed that all study recipients reached full donor chimerism by d+30 post-HCT (45). Therefore, functionally activated SARS-CoV-2-specific T cells, measured in the large majority of the HCT recipients (85%), originated from the donor graft at the start of prospective, longitudinal measurements. As expected, due to lymphopenia and immunosuppressive treatments (46), SARS-CoV-2-specific T cells in the recipients were significantly lower than in their HCT donors from the time of engraftment (~ +d30) to around +d100 post-transplant. As the early post-HCT immune deficit gradually improved, levels of donor-derived SARS-CoV-2-mediated cell immunity increased and closely mirrored those measured in HCT donors by 6 months post-HCT (**Figure 1**). This interesting finding strongly suggests that donor SARS-CoV-2-specific T cells are preserved and can actively proliferate post-HCT during immune reconstitution (12, 24, 43). Donor-derived SARS-CoV-2-specific CD4^+^ T-cell responses in the recipients were predominant compared to CD8^+^ T-cell responses, as typically observed in healthy individuals infected with COVID-19 (39). Several studies have shown that SARS-CoV-2-specific CD4^+^ T cells play a critical role in controlling and resolving a primary SARS-CoV-2 infection (38). Systemic immunosuppression for aGVHD, administered to the three recipients in whom SARS-CoV-2-specific T cells remained undetectable, emerged as the pivotal determinant for the lack of measurable cell-mediated adaptive responses, as reported in previous cases (47).

SARS-CoV-2-specific IFN-γ measured in the recipient cohort strongly correlated with levels of SARS-CoV-2-specific functionally activated T cells (**Figure 2**). These data are indicative that the donor-derived SARS-CoV-2-specific cellular immunity observed in the recipients was characterized by a polarized Th1 response, which has been described to be associated with protection from severe COVID-19 (42). Mobilization of a Th1 response has been shown to provide viral clearance and the establishment of long-lived SARS-CoV-2-specific CD4^+^ and CD8^+^ T cells (33).

The memory phenotype for both donor-derived S- and N-specific CD137^+^ T cells showed elevated frequencies of TCM, which can be home to lymph nodes, where they help B cells undergo affinity maturation (28, 33). TCM is also known for its ability to persist in circulation, proliferate, and give rise to effector T cells (48). Collectively, our data suggest that functionality of T-cell subset dominance and memory phenotypes of donor-derived SARS-CoV-2-specific T cells were preserved early post-HCT, and donor T-cell frequency was restored by 6 months post-HCT (12, 24, 43).

The gradual but continuous decline of the SARS-CoV-2 adaptive humoral response observed in the first 6 months post-HCT (**Figure 3**) is in sharp contrast with the concomitant growing trajectory of donor-derived SARS-CoV-2-specific cellular responses, measured in the recipients during the same post-HCT timeframe. This result supports the notion of delayed B-cell functional reconstitution and adaptive humoral immune recovery post-HCT (43, 49). SARS-CoV-2-specific humoral response profiles in patients who received a licensed COVID-19 vaccine or had SARS-CoV-2 infection pre-transplant were both of donor and recipient origin and therefore difficult to interpret. Nonetheless, they suggest that titers of NAb and S- and N-specific IgG were more durable than those in the recipients who received only donor-derived SARS-CoV-2 infection- or vaccine-stimulated humoral immunity. Hence, the pre-HCT COVID-19 licensed vaccination may increase post-HCT protection for the recipient if they are infected with SARS-CoV-2 (5). Further studies will be needed to confirm these preliminary observations, which were obtained in a very limited number of patients.

A powerful SARS-CoV-2 NAb response along with sustained levels of S- and subsequently N-specific functional T cells were measured in R86 (**Supplementary Figure 1S**), who developed COVID-19 infection post-HCT. The haplo donor had a confirmed history of COVID-19 infection, and the recipient did get vaccinated before the transplant. The recipient was SARS-CoV-2-free after 35 days, and the favorable outcome in this patient may have been linked both to the pre-HCT vaccination status of the recipient and the transfer of SARS-CoV-2-specific adaptive immunity from the haplo donor to the recipient, which included the immune transfer of Nab and S- and N-specific CD8^+^ and CD4^+^ T cells. Interestingly, both patients (R86 and R88) who survived post-HCT COVID-19 infection received a graft from a haplo donor and were still able to develop a SARS-CoV-2-specific T-cell response. Haploidentical stem cell transplantation using high-dose PTCy to prevent aGVHD and allow engraftment is increasingly used in patients lacking suitably matched donors (50, 51). Our finding of a measurable and functional T-cell response early post-HCT is in agreement with recent work indicating that PTCy neither induces pan-T-cell depletion nor eliminates alloreactive T cells (52).

In the patients who received BNT162b2 mRNA COVID-19 vaccination post-HCT, increases in NAb titers followed the surge of donor-derived SARS-CoV-2-specific CD4^+^ T cells (47, 53). This finding indicates that the CD4^+^ T-cell proliferation may have stimulated the vigorous SARS-CoV-2-specific adaptive humoral immunity, observed in these recipients. The critical role of CD4^+^ T cells (54) in promoting robust, long-lived SARS-CoV-2-specific antibody levels and in response to mRNA vaccines has been shown, including in HCT and cellular therapy recipients, in whom COVID-19 vaccines are not precluded even when B-cell aplasia occurs (55).

In conclusion, the current study outcome indicates that functional donor SARS-CoV-2 T cells are transferred to recipients undergoing moderate immunosuppressive regimens (12, 26). This adaptive immunity persists, expands, promotes, and strengthens functional vaccine responses early post-HCT and likely mitigates SARS-CoV-2 infection in the recipient.

Limitations of the study include the clinical heterogenicity of the D/R pairs and the small sample size of the prospectively analyzed recipient cohort. Moreover, PBMC from six MUD of the 15 recipients analyzed for the SARS-CoV-2 cellular immune response was not available. Future prospective studies in different transplant settings, with diverse immunosuppressive and ablative conditioning regimens, will provide greater clarity on the impact of SARS-CoV-2 donor-graft immune transfer. Such clinical studies can constitute a critical and essential step toward improving the remarkably poor recovery from COVID-19 observed in the HCT setting. They can also pave the way to identify novel vaccination strategies and evaluate further clinical approaches for augmenting protective immunity.

## Data availability statement

The raw data supporting the conclusions of this article will be made available by the authors, without undue reservation.

## Ethics statement

The trial was approved by the City of Hope (COH) and the National Marrow Donor Program (NMDP) institutional review boards (IRBs) and was undertaken in accordance with Good Clinical Practice guidelines and the Declaration of Helsinki. The patients/participants provided their written informed consent to participate in this study.

## Author contributions

CLR, MAM, SJF and DJD designed the study. HA, SO, IdA, VAP, IbA, MR, AMA, ASS, GM, SSD, RN, SJF, MAM treated the patients. YP, QZ, KG, ML, JL, TK, SOF, and M-AG performed specimen processing and immunological assays. CLR, FC, and YP analyzed the immune monitoring data. CR wrote the initial manuscript. All authors approved the final version.
