# Supplementary material for: Functional SARS-CoV-2-specific T cells of donor origin in allogeneic stem cell transplant recipients of a T-cell-replete infusion: A prospective observational study
Source: Front Immunol. 2023 Mar 3;14:1114131. doi: 10.3389/fimmu.2023.1114131 (PMC10020189; doi:10.3389/fimmu.2023.1114131)
Supplement: Supplementary file 1 [file DataSheet_1.docx]

**Supplementary material**

Functional SARS-CoV-2-specific T cells of donor origin in allogeneic stem cell transplant recipients of a T cell replete infusion: a prospective observational study

Corinna La Rosa, Flavia Chiuppesi, Yoonsuh Park, et al.

**Material and methods**

*4-1BB (CD137) and memory phenotypes staining with gating strategy.* Spike (S)- and Nucleocapsid (N)-specific T cell populations were identified by expression of 4-1BB (CD137), and four memory populations were defined according to the expression of CD28 and CD45RA. Peripheral blood mononuclear cells (PBMC) derived from blood specimens, collected at each time point were divided into three aliquots and stimulated for 24 hours with S-15mer megapool (kindly provided by A. Grifoni and A. Sette, La Jolla Institute for Immunology) (1), N peptide pools (GenScript), or medium (background control) as previously detailed (2). Cells were then stained with CD3 (PerCP Cy5.5), CD8 (V450), CD4 (PE Cy7), CD137 (APC), CD28 (BB5), and CD45RA (PE) antibodies (BD Biosciences). For each sample ~1x10^6^ PBMC were routinely used, and the bound fluorescent labels were measured using longitudinal multiparameter (six colors) fluorescence-activated cytometry. At least 200,000 events/sample were acquired on a Gallios™ flow cytometer and data analysis were performed with Kaluza software (Beckman Coulter, Brea, CA, USA). A primary gate was set on lymphocytes by use of forward and side scatter; secondary gates were set on CD3^+^ and then on CD3^+^ CD8^+^ or CD3^+^ CD4^+^ T cells. CD3^+^ CD8^+^ CD137^+^ or CD3^+^ CD4^+^ CD137^+^ T cells were expressed as a percentage of the respective reference population. When either SARS-CoV-2-specific CD137^+^ T cell populations were ≥0.2%, a further analysis for CD28 and CD45RA memory membrane markers was feasible (3). CD28 and CD45RA memory membrane marker expression in the CD3^+^ population was used as positive control to define the memory phenotype gating, and CD137^+^ T cells were divided in four memory subsets. Figure **1S** shows the gating strategy for S-specific CD3^+^ CD4^+^ T cells and relative memory phenotype subsets. CD45RA^+^ CD28^+^ cells were classified as naïve, CD45RA^-^ CD28^+^ cells as central memory (TCM), CD45RA^-^ CD28^-^ cells effector T cells (TEM) and CD45RA^+^ CD28^-^ effector “revertant” T cells, re-expressing the RA isoform of the CD45 surface marker (TEMRA)(4;5). Table **1S** reports the frequencies of S- and N-specific CD3^+^ CD4^+^ CD137^+^ T cells and memory subsets, which were measured in the 18 study HCT D/R pairs.

**Table 1S**. Memory phenotypes of Spike- and Nucleocapsid-specific CD3^+^CD4^+^CD137^+^ T cell responses

|  |  | Spike | | | | | Nucleocapsid | | | | |
| --- | --- | --- | --- | --- | --- | --- | --- | --- | --- | --- | --- |
| D/R | **Day**^1^ | **CD137%** | **TEMRA** | **TEM** | **Naïve** | **TCM** | **CD137%** | **TEMRA** | **TEM** | **Naive** | **TCM** |
| MUD06 | pre-HCT | 0.20 | 0.00 | 0.00 | 22.01 | 77.99 | 0.03 |  |  |  |  |
| R06 | 30 | 0.13 |  |  |  |  | 0.03 |  |  |  |  |
| R06 | 60 | 0.03 |  |  |  |  | 0.02 |  |  |  |  |
| R06 | 90 | 0.02 |  |  |  |  | 0.02 |  |  |  |  |
| R06 | 120 | 0.07 |  |  |  |  | 0.02 |  |  |  |  |
| R06 | 150 | 0.35 | 0.00 | 0.00 | 5.71 | 94.29 | 0.02 |  |  |  |  |
| R06 | 180 | 0.08 |  |  |  |  | 0.02 |  |  |  |  |
| MUD019 | pre-HCT | NA |  |  |  |  | NA |  |  |  |  |
| R019 | 30 | 0.02 |  |  |  |  | 0.02 |  |  |  |  |
| R019 | 60 | 0.04 |  |  |  |  | 0.02 |  |  |  |  |
| R019 | 90 | 0.02 |  |  |  |  | 0.02 |  |  |  |  |
| R019 | 120 | 0.11 |  |  |  |  | 0.02 |  |  |  |  |
| R019 | 150 | 0.02 |  |  |  |  | 0.02 |  |  |  |  |
| R019 | 180 | 0.02 |  |  |  |  | 0.02 |  |  |  |  |
| MUD029 | pre-HCT | 0.05 |  |  |  |  | 0.02 |  |  |  |  |
| R029 | 30 | 0.08 |  |  |  |  | 0.02 |  |  |  |  |
| R029 | 60 | 0.28 | 0.00 | 0.00 | 1.45 | 98.55 | 0.02 |  |  |  |  |
| R029 | 90 | 0.51 | 0.00 | 0.00 | 2.60 | 97.4 | 0.20 | 0.00 | 0.00 | 3.45 | 96.55 |
| R029 | 120 | 1.68 | 0.00 | 0.00 | 0.50 | 99.5 | 0.33 | 0.00 | 0.69 | 2.07 | 97.24 |
| R029 | 150 | 0.67 | 0.00 | 0.54 | 2.70 | 96.76 | 0.02 |  |  |  |  |
| R029 | 180 | 0.98 | 0.00 | 0.29 | 1.46 | 98.25 | 0.22 | 0.00 | 2.08 | 3.13 | 94.79 |
| MUD033 | pre-HCT | NA |  |  |  |  | NA |  |  |  |  |
| R033 | 30 | 0.41 | 0.00 | 0.00 | 13.85 | 86.15 | 0.03 |  |  |  |  |
| R033 | 60 | 0.57 | 0.00 | 0.00 | 5.12 | 94.88 | 0.06 |  |  |  |  |
| R033 | 90 | 0.20 | 0.00 | 0.00 | 16.67 | 83.33 | 0.03 |  |  |  |  |
| R033 | 120 | 0.94 | 0.00 | 0.00 | 1.89 | 98.11 | 0.20 | 0.00 | 0.00 | 6.94 | 93.06 |
| R033 | 150 | 0.47 | 0.00 | 0.00 | 0.83 | 99.17 | 0.38 | 0.00 | 0.00 | 4.90 | 95.1 |
| R033 | 180 | 0.43 | 0.00 | 0.00 | 5.62 | 94.38 | 0.02 |  |  |  |  |
| MUD036^2^ | pre-HCT | NA |  |  |  |  | NA |  |  |  |  |
| R036 | 30 | ND |  |  |  |  | ND |  |  |  |  |
| R036 | 60 | 0.02 |  |  |  |  | 0.02 |  |  |  |  |
| R036 | 90 | 0.02 |  |  |  |  | 0.02 |  |  |  |  |
| R036 | 120† | NV |  |  |  |  | NV |  |  |  |  |
| R036 | 150 |  |  |  |  |  |  |  |  |  |  |
| R036 | 180 |  |  |  |  |  |  |  |  |  |  |
| MUD040^2^ | pre-HCT | NA |  |  |  |  | NA |  |  |  |  |
| R040 | 30 | ND |  |  |  |  | ND |  |  |  |  |
| R040 | 60 | 0.02 |  |  |  |  | 0.02 |  |  |  |  |
| R040 | 90 | 0.02 |  |  |  |  | 0.02 |  |  |  |  |
| R040 | 120 | 0.02 |  |  |  |  | 0.02 |  |  |  |  |
| R040 | 150 | 0.02 |  |  |  |  | 0.02 |  |  |  |  |
| R040 | 180 | 0.02 |  |  |  |  | 0.02 |  |  |  |  |
| MUD041 | pre-HCT | NA |  |  |  |  | NA |  |  |  |  |
| R041 | 30 | 0.02 |  |  |  |  | 0.02 |  |  |  |  |
| R041 | 60 | 0.04 |  |  |  |  | 0.04 |  |  |  |  |
| R041 | 90 | 0.04 |  |  |  |  | 0.02 |  |  |  |  |
| R041 | 120 | 0.20 | 0.00 | 1.59 | 3.17 | 95.24 | 0.02 |  |  |  |  |
| R041 | 150 | 0.06 |  |  |  |  | 0.02 |  |  |  |  |
| R041 | 180 | 0.02 |  |  |  |  | 0.08 |  |  |  |  |
| MUD046 | pre-HCT | NA |  |  |  |  | NA |  |  |  |  |
| R046 | 30 | 0.05 |  |  |  |  | 0.07 |  |  |  |  |
| R046 | 60 | 0.04 |  |  |  |  | 0.02 |  |  |  |  |
| R046 | 90 | 0.02 |  |  |  |  | 0.05 |  |  |  |  |
| R046 | 120 | 0.20 | 0.00 | 0.00 | 4.44 | 95.56 | 0.08 |  |  |  |  |
| R046 | 150 | 0.61 | 0.28 | 0.28 | 2.50 | 96.94 | 0.10 |  |  |  |  |
| R046 | 180 | 0.21 | 0.00 | 0.00 | 4.00 | 96.00 | 0.02 |  |  |  |  |
| MUD049^2^ | pre-HCT | 0.09 |  |  |  |  | 0.20 | 0.00 | 0.00 | 4.00 | 96.00 |
| R049 | 30 | 0.02 |  |  |  |  | 0.02 |  |  |  |  |
| R049 | 60 | 0.02 |  |  |  |  | 0.02 |  |  |  |  |
| R049 | 90† |  |  |  |  |  |  |  |  |  |  |
| R049 | 120 |  |  |  |  |  |  |  |  |  |  |
| R049 | 150 |  |  |  |  |  |  |  |  |  |  |
| R049 | 180 |  |  |  |  |  |  |  |  |  |  |
| MUD050 | pre-HCT | NA |  |  |  |  | NA |  |  |  |  |
| R050 | 30 | 0.02 |  |  |  |  | 0.02 |  |  |  |  |
| R050 | 60 | 0.02 |  |  |  |  | 0.02 |  |  |  |  |
| R050 | 90 | 0.02 |  |  |  |  | 0.02 |  |  |  |  |
| R050 | 120 | 0.06 |  |  |  |  | 0.02 |  |  |  |  |
| R050 | 150 | 0.04 |  |  |  |  | 0.02 |  |  |  |  |
| R050 | 180 | 0.06 |  |  |  |  | 0.02 |  |  |  |  |
| MUD053 | pre-HCT | NA |  |  |  |  | NA |  |  |  |  |
| R053 | 30 | 0.02 |  |  |  |  | 0.02 |  |  |  |  |
| R053 | 60 | 0.02 |  |  |  |  | 0.05 |  |  |  |  |
| R053 | 90 | 0.02 |  |  |  |  | 0.02 |  |  |  |  |
| R053 | 120 | 0.03 |  |  |  |  | 0.02 |  |  |  |  |
| R053 | 150 | 0.04 |  |  |  |  | 0.06 |  |  |  |  |
| R053 | 180 | 0.22 | 0.00 | 0.00 | 6.45 | 93.55 | 0.26 | 0.00 | 0.00 | 3.00 | 97.00 |
| MUD054 | pre-HCT | 0.24 | 0.43 | 0.01 | 22.17 | 77.39 | 0.05 |  |  |  |  |
| R054 | 30 | 0.07 |  |  |  |  | 0.05 |  |  |  |  |
| R054 | 60 | NV |  |  |  |  | NV |  |  |  |  |
| R054 | 90 | 0.29 | 0.00 | 0.00 | 11.43 | 88.57 | 0.20 | 0.00 | 0.00 | 8.89 | 91.11 |
| R054 | 120 | 0.11 |  |  |  |  | 0.09 |  |  |  |  |
| R054 | 150 | 0.49 | 0.00 | 0.00 | 4.39 | 95.61 | 0.20 | 0.52 | 0.00 | 5.24 | 94.24 |
| R054 | 180 | 0.70 | 0.00 | 0.00 | 3.32 | 96.68 | 0.09 |  |  |  |  |
| Haplo062 | pre-HCT | 0.12 |  |  |  |  | 0.03 |  |  |  |  |
| R062 | 30 |  |  |  |  |  |  |  |  |  |  |
| R062 | 60 | 0.13 |  |  |  |  | 0.02 |  |  |  |  |
| R062 | 90 | 0.02 |  |  |  |  | 0.02 |  |  |  |  |
| R062 | 120 | 0.02 |  |  |  |  | 0.03 |  |  |  |  |
| R062 | 150 | 0.09 |  |  |  |  | 0.02 |  |  |  |  |
| R062 | 180 | 0.02 |  |  |  |  | 0.02 |  |  |  |  |
| MUD065 | pre-HCT | 0.76 | 0.00 | 0.00 | 13.9 | 86.1 | 0.32 | 0.45 | 0.00 | 6.82 | 92.73 |
| R065 | 30 | 0.06 |  |  |  |  | 0.02 |  |  |  |  |
| R065 | 60 | 0.04 |  |  |  |  | 0.02 |  |  |  |  |
| R065 | 90 | 0.02 |  |  |  |  | 0.02 |  |  |  |  |
| R065 | 120 | 0.02 |  |  |  |  | 0.04 |  |  |  |  |
| R065 | 150 | 0.09 |  |  |  |  | 0.08 |  |  |  |  |
| R065 | 180 | NV |  |  |  |  | NV |  |  |  |  |
| MUD078 | pre-HCT | 0.09 |  |  |  |  | 0.04 |  |  |  |  |
| R078 | 30 | 0.08 |  |  |  |  | 0.02 |  |  |  |  |
| R078 | 60 | 0.20 | 0.00 | 0.00 | 11.65 | 88.35 | 0.09 |  |  |  |  |
| R078 | 90 | NV |  |  |  |  | NV |  |  |  |  |
| R078 | 120 | 0.23 | 0.00 | 0.00 | 10.88 | 89.12 | 0.05 |  |  |  |  |
| R078 | 150 | LFU |  |  |  |  | LFU |  |  |  |  |
| R078 | 180 | LFU |  |  |  |  | LFU |  |  |  |  |
| Haplo086 | pre-HCT | 0.20 | 0.00 | 0.00 | 20.14 | 79.86 | 0.20 | 0.00 | 0.00 | 20.14 | 79.86 |
| R086 | 30 | 0.07 |  |  |  |  | 0.02 |  |  |  |  |
| R086 | 60 | 0.12 |  |  |  |  | 0.05 |  |  |  |  |
| R086 | 90 | 0.34 | 0.00 | 0.91 | 4.59 | 94.5 | 0.02 |  |  |  |  |
| R086 | 120 | 0.34 | 0.00 | 0.00 | 9.42 | 90.58 | 0.03 |  |  |  |  |
| R086 | 150 | 0.07 |  |  |  |  | 0.26 | 0.00 | 0.00 | 2.20 | 97.8 |
| R086 | 180 | 0.36 | 0.00 | 0.00 | 2.48 | 97.52 | 0.20 | 0.00 | 0.00 | 1.54 | 98.46 |
| Haplo087 | pre-HCT | 0.20 | 1.65 | 0.00 | 15.38 | 82.97 | 0.06 |  |  |  |  |
| R087 | 30 | 0.20 | 0.00 | 0.00 | 12.00 | 88.00 | 0.02 |  |  |  |  |
| R087 | 60 | 0.10 |  |  |  |  | 0.02 |  |  |  |  |
| R087 | 90 | 0.24 | 0.00 | 0.00 | 0.76 | 99.24 | 0.03 |  |  |  |  |
| R087 | 120 | 0.10 |  |  |  |  | 0.05 |  |  |  |  |
| R087 | 150 | 0.10 |  |  |  |  | 0.05 |  |  |  |  |
| R087 | 180 | 0.12 |  |  |  |  | 0.05 |  |  |  |  |
| Haplo088 | pre-HCT | 0.34 | 0.20 | 0.59 | 10.82 | 88.39 | 0.11 |  |  |  |  |
| R088 | 30 | NV |  |  |  |  | NV |  |  |  |  |
| R088 | 60 | NV |  |  |  |  | NV |  |  |  |  |
| R088 | 90 | 0.07 |  |  |  |  | 0.18 |  |  |  |  |
| R088 | 120 | NV |  |  |  |  | NV |  |  |  |  |
| R088 | 150 | NV |  |  |  |  | NV |  |  |  |  |
| R088 | 180 | 0.15 |  |  |  |  | 0.17 |  |  |  |  |

^1^Day= day post-hematopoietic stem cell transplant (HCT); ^2^Not included in **Figure 1** analyses since Spike- and Nucleocapsid-specific T cells were consistently 0.02% (assay detection limit).

D/R= HCT donor/recipient pair; MUD= matched unrelated donor; Haplo= Haploidentical donor; Pre-HCT= single blood draw up to 30 days before graft transfer to the HCT recipient; CD137%= percentages of CD3^+^CD4^+^CD137^+^ T cell responses specific for either Spike or Nucleocapsid, as indicated on the Table top row; TEMRA= percentages of CD45RA^+^ CD28^-^ effector “revertant” T cells, re-expressing the RA isoform of the CD45 surface marker; TEM= percentages of CD45RA^-^ CD28^-^ effector T cells; Naïve= percentages of CD45RA^+^ CD28^+^ naïve T cells; TCM= percentages of CD45RA^-^ CD28^+^ central memory T cells; NA= peripheral blood mononuclear cells (PBMC) not available since MUD specimens were received >24 hours after collection; ND= blood not drawn since patient was enrolled into the study after day 30 post-HCT; NV=PBMC not viable; LFU=patient lost to follow-up (terminally ill patient, in hospice care); †=patient expired.

**Legend to Figures**

**Figure 1S. Identification of** **CD137^+^ T cells and memory phenotype subsets.** Gating strategy to identify Spike (S)-specific CD3^+^ CD4^+^ CD137^+^ T cells and relative memory phenotype percentages. Representative PBMC from R029 on day 150 post-hematopoietic stem cell transplant (HCT) were stimulated 24 hours with S-15mer megapool as detailed in **Materials and methods**. A primary lymphocyte gate (gate A, **plot 1**) was set on forward and side scatter (FS INT versus SS INT), and subsequent gates were set on the CD3^+^ (gate B, **plot 2**) and then on CD3^+^ CD4^+^ (gate K, **plot 5**) T cells. In histogram **plots 3 and 4,** CD45RA and CD28 expressions of the gated CD3^+^ populations were identified, which were used to define the CD137^+^ T cell memory subset gating of **plot 7**. CD3^+^ CD4^+^ CD137^+^ (gate C2, **plot 6**) T cells were expressed as a percentage of the respective reference population. **Plot 7** represents the frequency of cells of each memory phenotype within the total S-specific CD3^+^ CD4^+^ CD137^+^  T cell population, which was considered 100%. CD45RA^+^ CD28^+^ cells were classified as naïve (upper right quadrant), CD45RA^-^ CD28^+^ cells as central memory (TCM; lower right quadrant), CD45RA^-^ CD28^-^ cells as effectors (TEM, lower left quadrant) and CD45RA^+^ CD28^-^ effector “revertant” T cells, re-expressing the RA isoform of the CD45 surface marker (TEMRA, upper left quadrant).

**Figure 2S. SARS-CoV-2-specific T cells and neutralizing antibodies in post-HCT COVID-19 infected and post-HCT vaccinated recipients.** Levels of S- and Nucleocapsid (N)-specific CD137^+^CD3^+^CD4^+^ and CD137^+^CD3^+^CD4^+^ T cells/μl (right y-axes) measured by multiparameter cytofluorimetry in available peripheral blood mononuclear cells (PBMC); and SARS-CoV-2- neutralizing antibodies, as serum dilution that neutralized 50% of the SARS-CoV-2 pseudo virus (PsV NT50, left y-axes). The plots show longitudinal SARS-CoV-2-specific adaptive humoral and cellular profiles in haploidentical donor/recipient (haplo D/R) HCT pair 86 (upper left plot) and 88 (upper right plot), and in matched unrelated donor (MUD)/R pairs 29 (lower left plot) and 46 (lower right plot). SARS-CoV-2 and syringe symbols, approximate post-HCT day of COVID-19 infection and BNT162b2 mRNA COVID-19 vaccination respectively (exact days are reported in **Results: Characteristics of the study population**).

**Figure 3S. Nucleocapsid specific IgG in HCT donors.** N-specific IgG in the 18 study HCT donors who either had history of COVID-19 (N=5) or were vaccinated (N=13). N-specific IgG were quantified using indirect ELISA and are expressed on the log_10_ y axes, as Endpoint titers. Box plots extend from the 25th to the 75th percentiles, median values are shown as a line, whiskers extend from minimum to maximum values. Individual values are superimposed. Reported on the histogram plot is the statistical testing results using Mann-Whitney test. Dashed line represents the lower limit of quantification.

**Figure 1S. Gating strategy for CD137^+^ and memory phenotype assays**


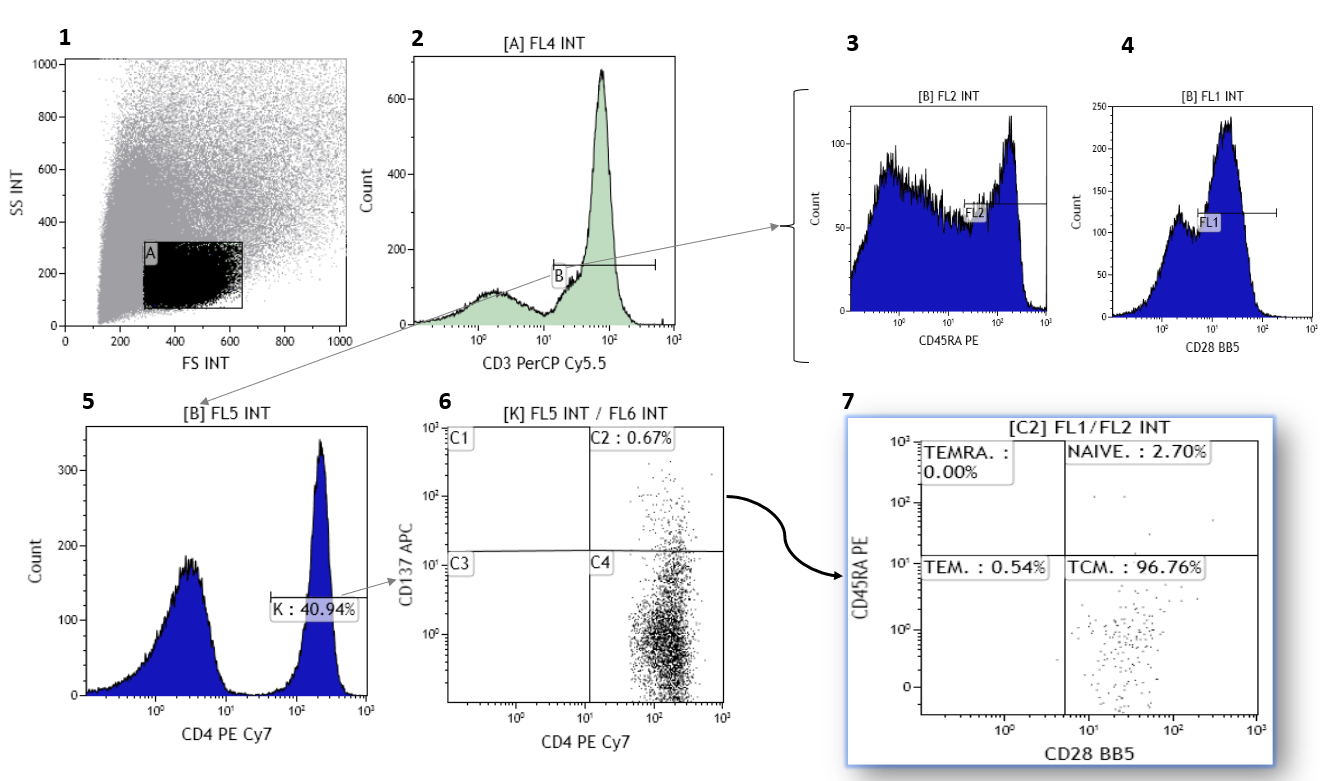


**Figure 2S. Recipients who developed COVID-19 or were vaccinated post-HCT**


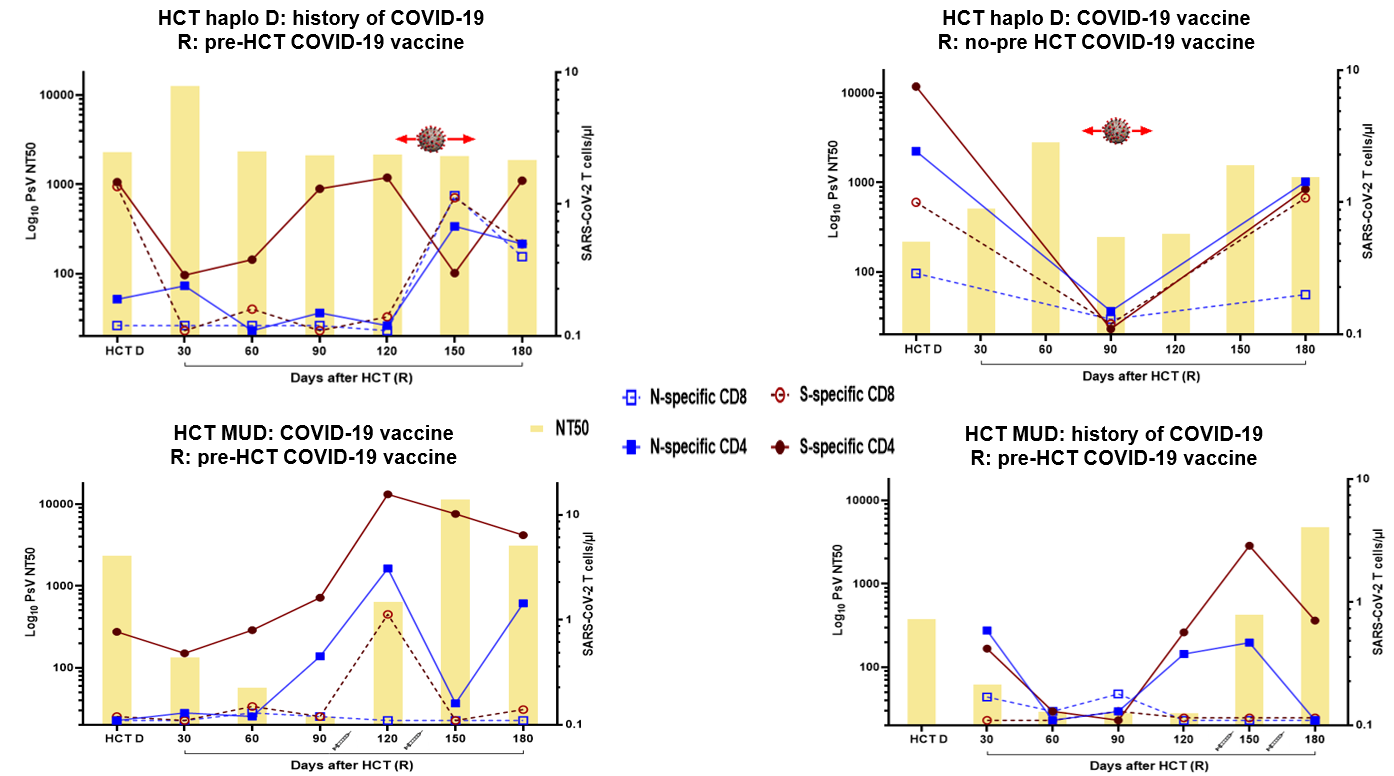


**Figure 3S. Nucleocapsid-specific IgG in HCT donors with history of COVID-19 or COVID-19 vaccination**


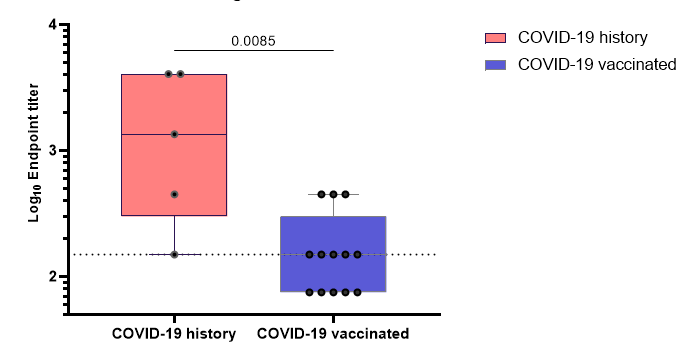


**References**

1. Tarke, A., Sidney, J., Kidd, C.K., Dan, J.M., Ramirez, S.I., Yu, E.D., Mateus, J., Da Silva Antunes, R., Moore, E., Rubiro, P., Methot, N., Phillips, E., Mallal, S., Frazier, A., Rawlings, S.A., Greenbaum, J.A., Peters, B., Smith, D.M., Crotty, S., Weiskopf, D., Grifoni, A., and Sette, A. (2021). Comprehensive analysis of T cell immunodominance and immunoprevalence of SARS-CoV-2 epitopes in COVID-19 cases. *Cell Rep Med* 2**,** 100204.

2. Chiuppesi, F., Zaia, J.A., Frankel, P.H., Stan, R., Drake, J., Williams, B., Acosta, A.M., Francis, K., Taplitz, R.A., Dickter, J.K., Dadwal, S., Puing, A.G., Nanayakkara, D.D., Ash, P., Cui, Y., Contreras, H., La Rosa, C., Tiemann, K., Park, Y., Medina, J., Iniguez, A., Zhou, Q., Karpinski, V., Johnson, D., Faircloth, K., Kaltcheva, T., Nguyen, J., Kha, M., Nguyen, V.H., Francisco, S.O., Grifoni, A., Wong, A., Sette, A., Wussow, F., and Diamond, D.J. (2022). Safety and immunogenicity of a synthetic multiantigen modified vaccinia virus Ankara-based COVID-19 vaccine (COH04S1): an open-label and randomised, phase 1 trial. *Lancet Microbe* 3**,** e252-e264.

3. La Rosa, C., Longmate, J., Martinez, J., Zhou, Q., Kaltcheva, T.I., Tsai, W., Drake, J., Carroll, M., Wussow, F., Chiuppesi, F., Hardwick, N., Dadwal, S., Aldoss, I., Nakamura, R., Zaia, J.A., and Diamond, D.J. (2017). MVA vaccine encoding CMV antigens safely induces durable expansion of CMV-specific T cells in healthy adults. *Blood* 129**,** 114-125.

4. Aldoss, I., La Rosa, C., Baden, L.R., Longmate, J., Ariza-Heredia, E.J., Rida, W.N., Lingaraju, C.R., Zhou, Q., Martinez, J., Kaltcheva, T., Dagis, A., Hardwick, N., Issa, N.C., Farol, L., Nademanee, A., Al Malki, M.M., Forman, S., Nakamura, R., Diamond, D.J., and Group, T.V.S. (2020). Poxvirus Vectored Cytomegalovirus Vaccine to Prevent Cytomegalovirus Viremia in Transplant Recipients: A Phase 2, Randomized Clinical Trial. *Ann Intern Med* 172**,** 306-316.

5. Neidleman, J., Luo, X., Frouard, J., Xie, G., Gill, G., Stein, E.S., Mcgregor, M., Ma, T., George, A.F., Kosters, A., Greene, W.C., Vasquez, J., Ghosn, E., Lee, S., and Roan, N.R. (2020). SARS-CoV-2-Specific T Cells Exhibit Phenotypic Features of Helper Function, Lack of Terminal Differentiation, and High Proliferation Potential. *Cell Rep Med* 1**,** 100081.
